# γ-Aminobutyric Acid Imparts Partial Protection from Salt Stress Injury to Maize Seedlings by Improving Photosynthesis and Upregulating Osmoprotectants and Antioxidants

**DOI:** 10.1038/srep43609

**Published:** 2017-03-08

**Authors:** Yongchao Wang, Wanrong Gu, Yao Meng, Tenglong Xie, Lijie Li, Jing Li, Shi Wei

**Affiliations:** 1 College of Agriculture, Northeast Agricultural University, Harbin 150030, P.R. China; 2 The Observation Experiment Station of Ministry of Agriculture for Crop Cultivation Science in Northeast Area, Harbin 150030, P.R. China; 3 Heilongjiang Academy of Land Reclamation Sciences, Harbin 100030, P.R. China

## Abstract

γ-Aminobutyric acid (GABA) has high physiological activity in plant stress physiology. This study showed that the application of exogenous GABA by root drenching to moderately (MS, 150 mM salt concentration) and severely salt-stressed (SS, 300 mM salt concentration) plants significantly increased endogenous GABA concentration and improved maize seedling growth but decreased glutamate decarboxylase (GAD) activity compared with non-treated ones. Exogenous GABA alleviated damage to membranes, increased in proline and soluble sugar content in leaves, and reduced water loss. After the application of GABA, maize seedling leaves suffered less oxidative damage in terms of superoxide anion (O_2_^·−^) and malondialdehyde (MDA) content. GABA-treated MS and SS maize seedlings showed increased enzymatic antioxidant activity compared with that of untreated controls, and GABA-treated MS maize seedlings had a greater increase in enzymatic antioxidant activity than SS maize seedlings. Salt stress severely damaged cell function and inhibited photosynthesis, especially in SS maize seedlings. Exogenous GABA application could reduce the accumulation of harmful substances, help maintain cell morphology, and improve the function of cells during salt stress. These effects could reduce the damage to the photosynthetic system from salt stress and improve photosynthesis and chlorophyll fluorescence parameters. GABA enhanced the salt tolerance of maize seedlings.

Soil salinity, especially that caused by NaCl, causes considerable abiotic stress to plants and affects both irrigated and non-irrigated farmland. On a global scale, approximately 20% of cultivated land and nearly 50% of irrigated land is affected by high salinity. It has been estimated that approximately 30% of the available land will be lost within the next 25 years and up to 50% will be lost by 2050 due to soil salinity^1^.

Maize (*Zea mays L.*) is one of the most important cereal crops and is grown under a wide spectrum of soil and climatic conditions. Maize is an important C_4_ plant from the Poaceae family and is moderately sensitive to salt stress^2^,^3^. Salinity reduces shoot growth by suppressing leaf initiation and expansion, as well as internode growth, and by accelerating leaf abscission. Salt stress rapidly reduces the leaf growth rate^4^ due to a reduction in the number of elongating cells and/or the rate of cell elongation^5^. Salt stress may lead to membrane damage^6^, a reduction in leaf relative water content^7^,^8^, the denaturation of proteins, the accumulation of oxidizing substances^9^, the inactivation of enzymes and the decline of photosynthesis in maize. Maize plants undergo a variety of adaptations at the subcellular, cellular, and organ levels to grow successfully under salinity. Maize plants exhibit several adaptations, such as stomatal regulation, changes in hormonal balance, activation of the antioxidant defence system, osmotic adjustment, maintenance of tissue water content, and various mechanisms of toxic ion exclusion under salt stress^10^.

γ-Aminobutyric acid (GABA) is a non-protein four-carbon amino acid that is produced by glutamate decarboxylase from glutamic acid^11^. GABA is a valuable component of the free amino acid pool that is widely distributed in nature among prokaryotes and eukaryotes. However, Su *et al*.^12^ suggested that high levels of GABA induced by salt stress can result from polyamine degradation, indicating that polyamine can perform its functions via GABA formation under salt stress. Some reports showed that GABA accumulation under salt stress occurs through calcium-induced activation of glutamate decarboxylase (GAD)^13^ and that GABA may be related to plant resistance. Li *et al*. suggested that exogenous GABA could improve leaf photosynthesis and chlorophyll fluorescence parameters, increase antioxidant enzyme activity, and reduce malondialdehyde accumulation and relative conductivity in wheat^14^. GABA in plants probably plays a dual role as both a signalling molecule and a metabolite. Moreover, GABA can be involved in regulating cytosolic pH and acts as an osmoregulator^15^. GABA acts as signal that triggers gene expression in *Agrobacterium tumefaciens*^16^. All genes involved in the GABA shunt are upregulated in the cDNA library in *Fusarium graminearum* grown on a plant cell wall^17^.

Recently, the main study on the GABA shunt in plants indicated that it is linked to stress and stress signalling^18^. Some reports have shown that the GABA shunt may be associated with various physiological responses, including signalling, osmoregulation, response to a fungal elicitor, nitrogen metabolism, carbon fluxes in the tricarboxylic acid cycle, the regulation of cytosolic pH^19^, and protection against oxidative stress. How the GABA metabolism changes in response to salt stress is largely unknown^20^. Thus, the role of GABA in salt tolerance must be examined further.

In the present study, to further elucidate the protective effects of GABA and its regulatory role in maize seedlings under salt stress, we investigated GABA concentration in the leaf, the growth of maize seedlings, membrane damage and leaf relative water content, the activities of antioxidant enzymes, proline and soluble sugar contents and photosynthesis in GABA-treated and untreated maize seedlings. The aim of this study was to provide a theoretical basis for the application of GABA in maize under salt stress.

## Results

### γ-Aminobutyric acid (GABA) concentration in leaves

**Figure 1:**
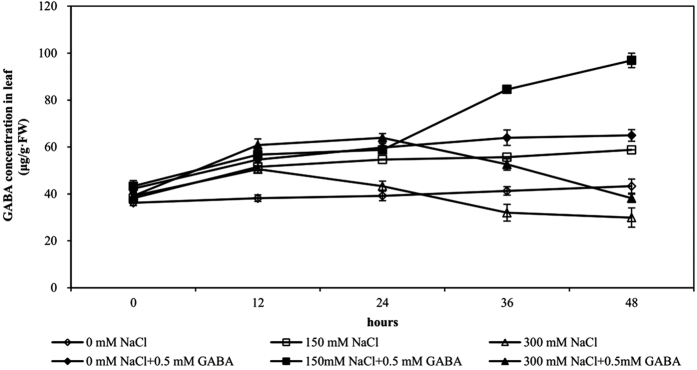
Endogenous γ-aminobutyric acid (GABA) concentration during the course of salt stress of varying intensity. Five-day-old plants were subjected to salt stress with or without 0.5 mM GABA. GABA concentration was measured during the stress period. Bars represent standard error of the mean (SE).

In control plants (0 mM NaCl), the concentration of endogenous GABA ranged between 36.18 and 43.30 μg g^−1^ fresh weight (FW) at 48 h (Fig. 1). In moderately salt-stressed (MS) plants (150 mM NaCl), the GABA concentration began to rise markedly after 36 h of stress and increased by 1.54-fold to reach 58.77 μg g^−1^ FW at 48 h. In the severely salt-stressed (SS) plants (300 mM NaCl), GABA concentration increased slightly after 12 h of stress but decreased markedly thereafter, falling below the GABA concentration of controls at 48 h of stress. The decrease in GABA concentration was associated with a drastic decrease in the growth of maize seedlings and an increase in oxidative damage to the cells. Exogenous application of GABA improved its endogenous levels by 1.65-fold in MS plants and 1.27-fold in SS plants after 48 h of stress.

### GABA application reduces GABA synthase activity

**Figure 2:**
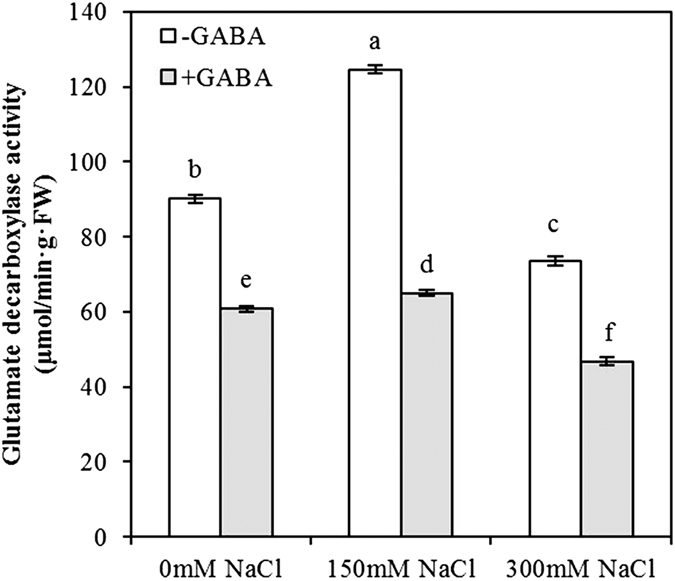
Glutamate decarboxylase activity at varying salt stress levels for 48 h in leaves of plants that were treated with 0.5 mM γ-aminobutyric acid (GABA) or untreated. Values are mean ± SE. Values with the same letters on the bars are not significantly different at P = 0.05 (LSD test).

Glutamate decarboxylase (GAD) activity was significantly affected by GABA and NaCl concentration; the interaction between GABA and NaCl concentration was significant (Table 1). GAD activity increased 1.38-fold in MS plants but decreased 1.23-fold in SS plants compared with that of controls. Compared to plants not treated with exogenous GABA, the GAD activity of GABA-treated plants was significantly reduced, by 32.59%, 47.79% and 36.29% in control, MS and SS treatments, respectively (Fig. 2).

### Maize phenotypes with or without treatment of GABA under salt stress

**Figure 3:**
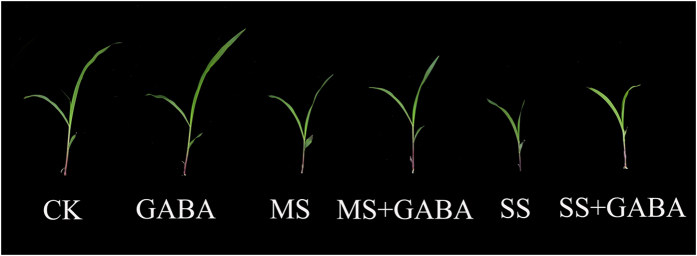
Phenotypes of maize with or without GABA treatment under salt stress.

Maize seedling plant height decreased during salt stress, especially in severely salt-stressed plants. Moreover, the leaf area and the seedling stalk height were significantly reduced under salt stress. Maize seedling growth was restored by applying exogenous GABA under salt stress, and exogenous GABA also increased the growth of non-stressed seedlings (Fig. 3).

### GABA application improves the growth of salt stress plants

Plant height, leaf area per plant, leaf fresh and dry weight per plant were significantly affected by GABA and NaCl concentration; the interaction between GABA and NaCl concentration was not significant (Table 1).

Growth was measured at 48 h of salt stress (Table 2). As the level of salt stress increased, the extent of inhibition increased and was linked to the reduction in endogenous GABA concentration. At 48 h, plant height was reduced by 27% in MS plants and 46% in SS plants compared with that of controls. Similarly, the leaf area per plant, leaf fresh weight per plant and leaf dry weight per plant were reduced by 26%, 43% and 37% in MS plants and 46%, 62% and 52% in SS plants, respectively. GABA application enhanced the plant height of MS plants by 25% and that of SS plants by 18%, and the leaf area per plant increased by 30% and 28%, respectively. GABA application increased the leaf fresh and dry weight by 51% and 52%, respectively, in MS plants.

### GABA reduces membrane damage, enhances leaf water content, and reduces damage to mitochondrial and chloroplast function in salt-stressed plants

Membrane damage, SPAD value, leaf relative water content (RWC) and cellular reduction ability were significantly affected by GABA and NaCl concentration. The interaction between GABA and NaCl concentration was not significant except for RWC (Table 1).

The damage to leaf membranes (measured as electrolyte leakage) increased by 31% in MS plants and 42% in SS plants compared with that in controls. GABA application reduced the membrane damage significantly under salt stress. The SPAD value decreased by 22% and 40% in MS and SS plants, respectively, compared with that of controls. GABA-treated plants, especially those grown at 150 mM NaCl, had significantly higher SPAD values.

The RWC of leaves decreased to 12% in MS plants and 24% in SS plants compared with that of controls. GABA-treated plants were able to maintain significantly greater RWC than non-treated plants under salt stress. Mitochondrial function was measured as cellular reduction ability using the 2,3,5-triphenyltetrazolium chloride (TTC) test and decreased to 15% in MS plants and 41% in SS plants. MS plants treated with GABA exhibited a 17% improvement in mitochondrial function (Table 3).

### GABA promotes the accumulation of the osmolytes proline and soluble sugar under salt stress

Proline (Pro) and soluble sugar contents were significantly affected by GABA and NaCl concentration; the interaction between GABA and NaCl concentration was significant (Table 1).

**Figure 4:**
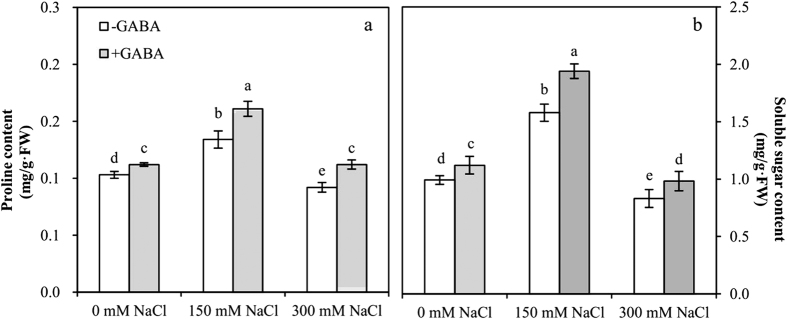
Proline (**a**) and soluble sugar (**b**) contents at varying salt stress for 48 h in the leaves of plants that were treated with 0.5 mM γ-aminobutyric acid (GABA) compared to untreated plants. Values are mean ± SE. Values with the same letters on the bars are not significantly different at P = 0.05 (LSD test).

In the leaves of MS plants, Pro exhibited a 1.3-fold accumulation, but in SS plants, a reduction in Pro content was found (Fig. 4a). There was an increase in Pro accumulation of 1.2-fold in GABA-treated MS plants and 1.2-fold in GABA-treated SS plants compared with that in plants grown without GABA treatment. Soluble sugar content increased 1.9-fold in MS plants and 1.2-fold in SS plants compared with that of controls. GABA application resulted in a significant increase in the soluble sugar content of MS and SS plants relative to untreated plants (Fig. 4b).

### GABA reduces oxidative damage to salt-stressed plants

Malonaldehyde (MDA) and superoxide anion (O_2_^·−^) contents were significantly affected by GABA and NaCl concentration; the interaction of O_2_^·−^ content between GABA and NaCl concentration was significant (Table 1).

**Figure 5:**
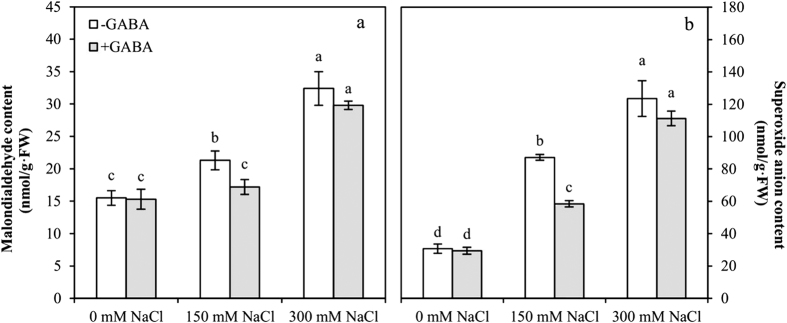
Malondialdehyde (**a**) and superoxide anion (**b**) content for growing at varying salt stress 48 h in leaves of plants that were treated with 0.5 mM γ-aminobutyric acid (GABA) or left untreated. Values are mean ± SE. Values with the same letters on the bars are not significantly different at P = 0.05 (LSD test).

Oxidative stress (MDA and O_2_^·−^ contents) increased rapidly in leaves with an increase in salt stress (Fig. 5). MDA and O_2_^·−^ contents increased 1.4-fold and 2.8-fold in MS plants, respectively, compared with those of the controls. In SS plants, MDA and O_2_^·−^ contents increased 2.1-fold and 4.0-fold, respectively, compared with those of the controls. GABA application significantly reduced the MDA and O_2_^·−^ contents of MS plants compared with those of the controls.

### Histochemical localization of O_2_^·−^ in maize leaves

**Figure 6:**
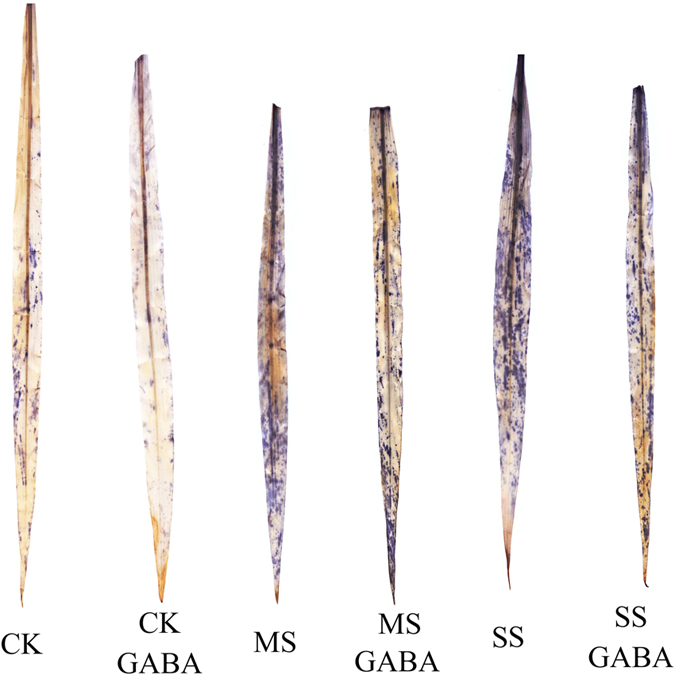
Histochemical localization of O_2_^·−^ in leaves of maize seedlings.

Histochemical staining was performed to localize O_2_^·−^ in the leaves of the maize seedlings (Fig. 6). A significant increase in the accumulation of O_2_^·−^, indicated by dark blue spots, was observed in the leaves of the maize seedlings in MS and SS plants compared with that in the controls. GABA application significantly reduced the number dark blue spots in MS and SS plants, indicating that the accumulation of superoxide anion was suppressed by applying exogenous GABA.

### GABA promotes the upregulation of antioxidant activity

Superoxide dismutase (SOD), peroxidase (POD), catalase (CAT) and ascorbate peroxidase (APX) activities were significantly affected by GABA and NaCl concentration; the interaction between GABA and NaCl concentration was significant (Table 1).

The activities of antioxidant enzymes such as SOD (transforms superoxide oxygen radicals into H_2_O_2_), POD and CAT (transforms H_2_O_2_ into water and oxygen), and APX (uses ascorbate to remove H_2_O_2_) were recorded in salt-stressed plants grown with or without GABA. SOD activity increased 2.4-fold in MS plants but decreased 1.7-fold in SS plants compared with that in the controls. POD activity also increased to a similar extent in MS plants (1.5-fold) compared with that in the controls but declined 1.5-fold in SS plants. The activity of SOD was significantly enhanced in GABA-treated MS and SS plants. The activity of POD was significantly enhanced in GABA-treated MS plants (Table 4).

CAT activity increased 1.1-fold in MS plants but decreased 1.4-fold in SS plants compared with that in the controls. APX activity increased 1.6-fold in MS plants but decreased 2.0-fold in SS plants compared to controls. The activities of the two enzymes were significantly higher (1.4–1.5-fold) in GABA-treated plants compared with that in those grown without GABA (Table 4).

### GABA promotes photosynthesis in salt stress

The net photosynthetic rate (P_n_), intercellular CO_2_ concentration (C_i_) and stomatal conductance (G_s_) were significantly affected by GABA and NaCl concentration, and the transpiration rate (trmmol) was significantly affected by NaCl concentration. The interaction between GABA and NaCl concentration was not significant (Table 1).

**Figure 7:**
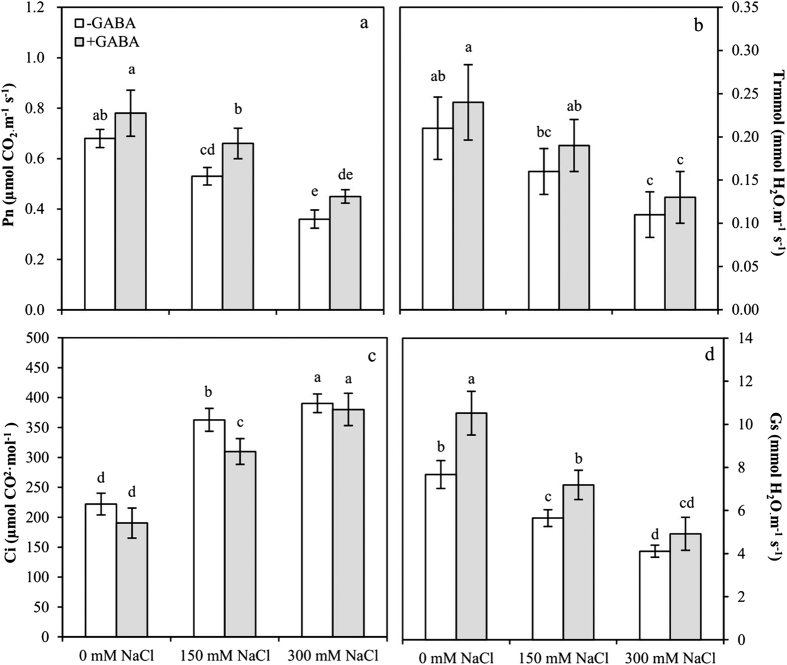
Net photosynthetic rate, P_n_ (**a**); transpiration rate, Trmmol (**b**); intercellular CO_2_ concentration, Ci (**c**); and stomatal conductance, Gs (**d**) for growing at varying salt stress at 48 h in leaves of plants that were treated with 0.5 mM γ-aminobutyric acid (GABA) or left untreated. Values are mean ± SE. Values with the same letters on the bars are not significantly different at P = 0.05 (LSD test).

P_n_ and trmmol decreased in the leaves with an increase in salt stress (Fig. 7a,b). P_n_ and trmmol decreased 1.3-fold and 1.3-fold in MS plants and decreased 1.9-fold and 1.9-fold in SS plants compared with that in the controls. GABA application caused an increase in P_n_ and trmmol compared with that in untreated plants. GABA-treated MS plants exhibited a significant increase in P_n_ (1.2-fold) compared to the untreated plants. C_i_ rose significantly in leaves during salt stress (Fig. 7c). C_i_ increased 1.6-fold in MS plants and 1.7-fold in SS plants compared with that in the controls. GABA application resulted in a decrease in C_i_ compared with that in untreated plants. GABA-treated MS plants exhibited a significant decline in C_i_ compared with that in untreated MS plants (0.8-fold). G_s_ decreased significantly in the leaves during salt stress (Fig. 7d). The G_s_ decreased 1.4-fold and 1.9-fold in MS and SS plants, respectively, compared with that in the controls. With GABA application, G_s_ increased compared with that in plants grown without it.

### Chlorophyll fluorescence

Primary fluorescence (F_0_), maximal fluorescence (F_m_), maximum quantum efficiency of Photosystem II (PSII) photochemistry (F_v_/F_m_) and electron transfer rate (ETR) were significantly affected by GABA and NaCl concentration. The interactions of F_m_ and ETR between GABA and NaCl concentration were not significant (Table 1).

**Figure 8:**
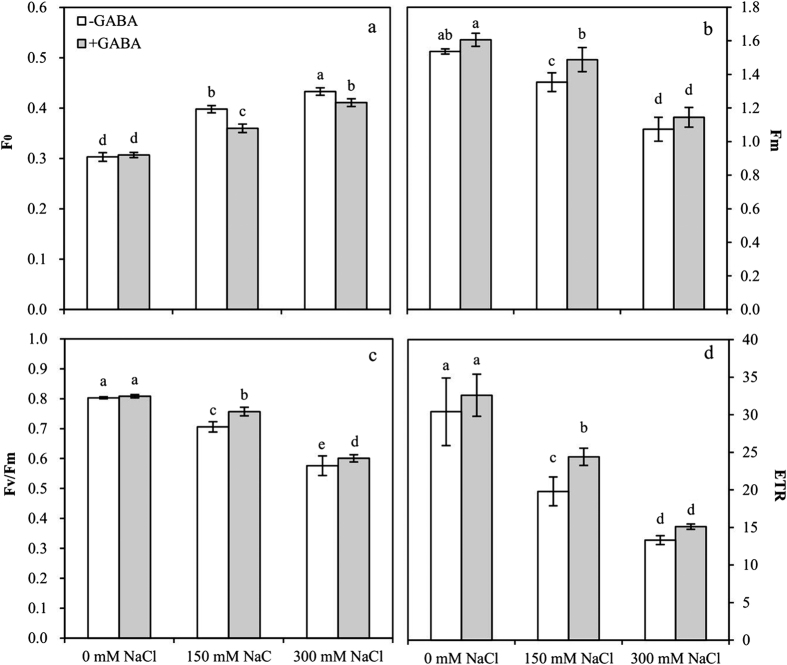
Primary fluorescence, F_0_ (**a**); maximal fluorescence, F_m_ (**b**); maximum quantum efficiency of PSII photochemistry, F_v_/F_m_ (**c**); and electron transfer rate, ETR (**d**) for plants grown at varying salt stress at 48 h in leaves of plants that were treated with 0.5 mM γ-aminobutyric acid (GABA) or left untreated. Values are mean ± SE. Values with the same letters on the bars are not significantly different at P = 0.05 (LSD test).

Chlorophyll fluorescence was measured for primary fluorescence (F_0_), maximal fluorescence (F_m_), maximum quantum efficiency of PSII photochemistry (F_v_/F_m_) and ETR (Fig. 8). The F_0_ increased 1.3-fold and 1.4-fold in MS and SS plants, respectively. There was a significant decline in F_0_ in GABA-treated MS and SS plants compared with those growing without GABA (Fig. 8a). Salt stress significantly decreased F_m_ in MS (11.8%) and SS (30.1%) plants compared with that in the controls. The F_m_ of GABA-treated MS plants significantly increased by 9.9% compared with that in untreated plants (Fig. 8b). Similarly, F_v_/F_m_ and ETR decreased by 12.1% and 34.9% and by 28.3% and 56.3% in MS and SS plants, respectively. The F_v_/F_m_ and ETR of GABA-treated MS plants increased by 7.2% and 18.8%, respectively, (Fig. 8c,d).

## Discussion

Salt stress is an important abiotic stress that seriously affects crop productivity and survival. The accumulation of γ-aminobutyric acid (GABA) is strongly induced by salt stress^21^. Endogenous GABA concentration increased in moderately salt-stressed (MS, 150 mM NaCl) maize seedlings but decreased sharply in severely salt-stressed (SS, 300 mM NaCl) maize seedlings. In a previous study, under salt stress, *A. halophytica* cells accumulated approximately 2-fold-higher levels of GABA compared with that in the controls. Our examination of GAD activity revealed that GAD activity significantly increased in MS plants but significantly decreased in SS plants compared with that in controls, and this trend was similar to endogenous GABA concentration. Therefore, the change in endogenous GABA concentration is associated with GAD activity, which is consistent with previous results, such as those reported by Boonburapong *et al*.^22^. We confirmed this finding with the exogenous application of 0.5 mM GABA, which resulted in a significant increase in endogenous GABA concentration in maize seedlings, consistent with a recent study by Xing *et al*.^23^. Moreover, an increase in exogenous GABA concentration was related to an increase in endogenous GABA concentration under chilling stress^24^. GABA is a non-protein four-carbon amino acid that can be absorbed directly by plants; this may be the reason that treatment with exogenous GABA can increase endogenous GABA concentration. Although exogenous GABA reduces GAD activity and affects endogenous GABA synthesis via the GABA shunt, the endogenous GABA concentration of GABA-treated plants was higher than that in untreated plants. A reasonable explanation is that exogenous GABA has a feedback regulation effect on GAD activity and has other GABA biosynthesis effects, e.g., a polyamine degradation process.

Salt stress may also displace calcium from plasma membrane binding sites, causing membrane leakiness as a primary cellular response to salt stress. Membrane leakiness increases the exudation of ions, causing an increase in leaf relative conductivity^25^. In maize, sodium is the principal toxic ion that interferes with potassium uptake and transport leading to a disturbance in stomatal modulation and causes water loss and necrosis^26^,^27^. Toxic levels of sodium in plant organs damage subcellular organelles, reducing cellular reduction ability and causing chlorophyll degradation before plant mortality^28^,^29^. Our results were similar with those of previous studies showing that salt stress increases membrane damage (relative conductivity) and decreases the SPAD value, relative leaf water content and cellular reduction ability in MS and SS maize seedlings. GABA application reduces the damage caused by salt stress on maize leaves, including a reduction of membrane damage and an increase in SPAD, relative leaf water content and cellular reduction ability in MS and SS plants. Compared with SS plants, MS plants had a better response to GABA under salt stress. In other stress conditions, such as heat or hypoxia, membrane integrity, chlorophyll content, and mitochondrial function were restored significantly by the application of GABA^30^. How GABA affected or restored these functions in salt-stressed cells still has to be explored and may be associated with an improvement of water status along with a reduction in oxidative damage to the membranes of organelles and cells.

Osmotic adjustment or osmoregulation is a key adaptation of plants at the cellular level to minimize the effects of salinity-induced drought stress. Soluble sugars, proline, glycine betaine, and trehalose are among the major osmolytes. Kaya *et al*. reported that proline accumulation increases in maize plants experiencing salt stress^7^. Likewise, Mansour *et al*. reported an increased accumulation of both proline and glycine betaine in maize under salt stress^31^. Our studies were similar to previous results showing that the accumulation of proline and soluble sugar significantly increases in MS plants but dramatically decreases in SS plants. The effect of salt stress on plants has two main phases: osmotic stress during the first phase and ion toxicity during the second phase^26^. In our results, MS treatment (150 mM NaCl) might be similar to the first phase of salt stress due to a lower ion concentration (sodium and chloride) and could respond to salt stress through self-regulating systems such as the accumulation of proline and soluble sugar. SS treatment (300 mM NaCl) might be similar to the second phase of salt stress, and ion toxicity was caused by a higher ion concentration, which affected normal regulatory functions and hindered the synthesis of proline and soluble sugar. GABA application increased the accumulation of proline and soluble sugar in MS and SS plants. MS plants had a better response to GABA regulation, accumulating more proline and soluble sugar than SS plants. These findings confirm previous results showing that applying GABA increased proline and soluble sugar contents during stress and improved the ability of plants to resist stressful environments^32^. The citric acid cycle is the main way of living organisms to obtain energy and it is a common metabolic pathway of sugar, fat and protein by which to be completely oxidized in the body. Succinic acid and α-ketoglutarate are important intermediate products of the citric acid cycle and can be generated by the GABA oxidation reaction. Exogenous GABA-induced increases in proline and soluble sugar contents might be indirectly associated with its participation in the citric acid cycle.

Salt stress-induced reactive oxygen species (ROS) generation leads to membrane damage and associated lipid peroxidation during stress^33^ and causes an excessive increase of malonaldehyde (MDA) content. MDA accumulation is considered an indicator of plant oxidative stress^34^. The antioxidase system, including SOD, POD, CAT and APX, plays an important role in ROS metabolism^35^. The activity of antioxidases in maize seedlings increased in salt stress, such as SOD, POD, CAT and APX^36^. Some reports also found that CAT activity decreased under salinity^37^, whereas SOD activity increased^38^. A similar result was found here, in which MDA and superoxide anion (O_2_^·−^) contents increased as the degree of salt stress increased. The activities of SOD, CAT, APX, and POD increased to reduce injury from oxidative stress in MS plants but decreased in SS plants. Maize plants facing salt stress employ a variety of accommodative mechanisms at the molecular level to resist the damaging effects of salinity stress. Of these, the upregulation and downregulation of many gene transcripts are important^39^. The expression of antioxidant defence genes is induced in maize to protect cells from salinity-induced oxidative damage. In maize, CAT activity increases due to the induction of mRNA accumulation in response to higher ROS levels under salt stress^10^. Compared with gene expression in the controls, the relative gene expressions of SOD, POD and APX increased but that of CAT decreased under salt stress in tomato seedlings. The relative gene expressions of APX and POD increased in 200 mM NaCl concentration but were lower than in 100 mM NaCl concentration^40^. Higher NaCl concentrations may hinder gene expression, resulting in a decline in enzymatic activity, which may be a reasonable interpretation of our results.

GABA application decreased MDA and O_2_^·−^ contents in MS and SS plants. GABA had a greater effect on MS plants than on SS plants. The plants that received GABA + NaCl exhibited higher antioxidant enzyme activity than those that received NaCl alone. GABA had a more profound effect on the antioxidant enzyme activity of MS plants. The results are similar to previous studies showing that MDA as an index of membrane injury was lower in the GABA+NaCl plants than in the plants treated with NaCl alone, which is consistent with higher antioxidant enzyme activity^41^. Similar results were reported in PEG-stressed plants by Vijayakumari and Jos^42^. The mechanism that allows GABA to induce the upregulation of antioxidative molecules has yet to be investigated. Previous studies indicate that GABA can act as a signalling molecule to activate some enzymes^43^ and can induce gene expression for nitrate uptake in *Brassica napus L*. Further studies of GABA found it to be protective, especially in the regulation of antioxidant activity^44^.

Photosynthesis is the most important process by which green plants convert solar energy to chemical energy in the form of organic compounds synthesized by the fixation of atmospheric carbon dioxide^8^. Salt stress hinders plant absorption of moisture and nutrients, causing malnutrition, and decreases chlorophyll content, thus inhibiting photosynthetic capacity^45^. NaCl inhibits photosynthetic capacity by decreasing CO_2_ fixation, stomatal conductance, and transpiration. In our studies, the net photosynthetic rate (P_n_), transpiration rate (Trmmol) and stomatal conductance (G_s_) decreased under salt stress in maize. Our finding that intercellular CO_2_ concentration (C_i_) increased was different from that reported by Wu *et al*.^46^. A reasonable explanation is that the photosynthetic capacity suffered damage from salt stress, resulting in the reduction of CO_2_ assimilation. PSII is considered to be the primary site of the photosynthetic apparatus injury during stress^47^. Injury to PSII can lead to a change in chlorophyll fluorescence. Therefore, chlorophyll fluorescence can be used as a powerful and reliable non-invasive method to assess changes in the function of PSII and to examine the primary photosynthetic processes under environmental stress conditions^48^. In our results, maximal fluorescence (F_m_), maximum quantum efficiency of PSII photochemistry (F_v_/F_m_) and ETR decreased and primary fluorescence (F_0_) increased. Those results are similar to those reported by Naumann *et al*.^49^. Salinity stress damages the oxygen-evolving complex and inhibits the quantum yield of PSII electron transport and photochemical efficiency^50^. Decreased PSII activity under salinity stress is considered the result of decreased excitation energy reaching PSII reaction centres (RCs) and changes in the pigment–protein complexes of thylakoid membranes^51^, which may be reasonable explanations for the observed photosynthetic capacity decline in salt stress.

Some reports found that plants that received GABA + NaCl had a higher P_n_ and G_s_ than those that received NaCl alone^52^ and that exogenous GABA enhanced Trmmol and C_i_ under varying degrees of salt stress^41^. The GABA + NaCl plants had higher F_v_/F_m_ and F_m_ under severe salinity compared with those of the plants that received NaCl alone^53^. In our study, exogenous GABA improved photosynthetic capacity; increased P_n_, Trmmol and G_s_; and decreased C_i_. Moreover, chlorophyll fluorescence improved in plants that received GABA + NaCl compared with that in those that received NaCl alone. Exogenous GABA can reduce the accumulation of harmful substances^54^, maintain cell morphology^55^, and improve the function of the cell in salt stress. These protective effects could protect photosystem II from salt stress damage and improve chlorophyll fluorescence parameters and increase the absorption of light energy and electron transfer. Eexogenous GABA can ease the damage caused by salt stress and increase leaf relative water content, thus improving trmmol and G_s_. Moreover, due to the improvement of cell function, P_n_ was enhanced. This effect improved the utilization of CO_2_ and decreased C_i_ in maize seedling leaves.

## Methods

### Materials

Maize seeds, “Zhengdan 958” (*Zea mays* L.), supplied by the Henan Academy of Agricultural Sciences, were used in this experiment. γ-Aminobutyric acid (GABA) (99% purity, CAS No. 56-12-2) was purchased from Sigma-Aldrich Co., Ltd. (St. Louis, MO, USA).

### Growth conditions and treatment

Maize seeds were sterilized with 0.1% mercuric chloride for 10 min and subsequently washed thoroughly three times with distilled water after overnight imbibition. Petri dishes (15 cm diameter) lined with moist filter paper were used for seed germination after high-temperature (120 °C) sterilization for 30 min. Maize seeds were grown in glasshouse conditions for 5 days at 28/20 °C day/night temperature, relative humidity (RH) = 65–70%, and 14/10 h light/dark photoperiod under natural light until the emergence of shoots and roots. The light intensity during the day cycle was maintained at a minimum of 400 μmol m^−2^ s^−1^ using supplemented light (Philips high-pressure sodium lamps). The moist filter paper was replaced every day. On the fifth day, forty maize seedlings of uniform growth were selected and transferred to one plastic box (length × width × height: 30 × 20 × 7 cm) containing modified 1/2 Hoagland solution (pH = 6.5) and fixed in perforated foam board by an opening sponge.

At the three-leaf stage, maize seedlings growing in the plastic box were subjected to the following treatments by root drenching: (1) 0 mM NaCl +0 mM GABA (CK), (2) 150 mM NaCl +0 mM GABA (moderate stress, MS), (3) 300 mM NaCl +0 mM GABA (severe stress, SS), (4) 0 mM NaCl +0.5 mM GABA (GABA), (5) 150 mM NaCl +0.5 mM GABA (MS + GABA), and (6) 300 mM NaCl +0.5 mM GABA (SS + GABA). Maize seedlings in the GABA, MS + GABA and SS + GABA treatment groups were pretreated with 0.5 mM GABA nutrient solution for 24 h before stress. Each treatment was repeated three times, and forty maize seedlings from one plastic box were considered a repeat of each treatment. All plastic boxes were arranged completely randomly. Maize seedlings were grown under glasshouse conditions at 28/20 °C day/night temperature, 14/10 h light/dark photoperiod under natural light. The light intensity during the day cycle was maintained to a minimum of 400 μmol m^−2^ s^−1^ using supplemented light (Philips high-pressure sodium lamps). The GABA concentration was selected after using a range of GABA concentrations (0.25–2 mM) in a preliminary growth experiment (data are provided in the Supplementary Dataset). The best GABA concentration was chosen on the basis of improved growth of the plants in a nutrient solution containing 150 mM NaCl. The plants (the second leaf) were assessed after 48 h of exposure to salt stress in the presence or absence of GABA for endogenous GABA content, growth, stress injury to membranes, SPAD value, chlorophyll fluorescence, photosynthetic rate, osmolytes, oxidative stress, and antioxidants as follows.

### Endogenous GABA concentration

GABA content was measured according to the method of Saito *et al*.^56^. A total of 0.5 g of fresh leaves was triturated in liquid nitrogen; 3 ml 70 mmol/l LaCl_3_ solution (φ = 80% methanol solution preparation) was added to the grated leaves while they were homogenized. The homogenate was transferred into centrifuge tubes and extracted overnight at 4 °C. The samples were centrifuged (Hermle Labortechnik GmbH) at 3000 r/min for 10 min, 1 ml supernatant was transferred to a 1.5 ml centrifuge tube, and 210 μl mol/l KOH was added to a 1.5 ml centrifuge tube and mixed for 10 min. Afterward, the samples were centrifuged at 12,000 × g for 10 min; the supernatant was removed and evaporated until dry in an 80 °C water bath. After the residue was dissolved by a buffer solution of 0.5 ml 0.5 mol/l K_2_HPO_4_-KH_2_PO_4_, pH 8.6, GABA content was measured with enzymes.

### Plant growth

Plant height was measured with a metre stick (0.1 cm minimum scale). Plant leaves were cut and weighed to determine fresh weight. Leaves were dried for 20 min at 105 °C, then dried for 40 min to constant weight at 80 °C, and the dry weight was measured. The leaf area of each plant was measured from fresh leaves using a LICOR LA-3100 planimeter (LI-COR, Inc.)^57^. Maize seedling root date can be checked in Supplementary Data.

### Stress injury

Samples of 0.5 g of fresh leaves were repeatedly washed with deionized water, cut into pieces (length × width: 1.0 × 0.5 cm) and put into test tubes with a plug. A volume of 10 ml deionized water was put into the test tubes, vacuumized for 10 min, vibrated, covered with a plug, and placed at room temperature for 30 min. Solution conductivity was measured with a DDS-11A-type (Shanghai INESA Scientific Instrument Co., Ltd) conductivity metre (E_1_); then, the test tubes were placed into boiling water for 15 min and the solution conductivity (E_2_) was measured after cooling. The conductivity of deionized water was E_0_. Membrane permeability P (%) = [(E_1_ − E_0_)/(E_2_ − E_0_)]^58^.

Cellular reduction ability: 0.1 g plant tissue was put into a 10 ml beaker; 5 ml 0.4% TTC solution and 5 ml phosphate buffer solution were added to the beaker. The sample was placed in the dark for 2 h at 37 °C, and then 2 ml 1 mol/l sulfuric acid was added to terminate the reaction. Plant tissue was taken out and ground with 3 ml ethyl acetate after the water was drained to extract methyl hydrazone. Red extracts and ethyl acetate that were used to rinse off the residue were transferred to a test tube to ensure that the total amount of ethyl acetate was 10 ml. The absorbance of the extract was read at 485 nm using a spectrophotometer.

Fresh leaves were cut and weighed to determine their FW. Whole leaves were immersed in distilled water and keep in dark for 12 h at 4 °C. Subsequently, the leaves were removed from deionized water, the surface moisture was removed, and the leaves were weighed for turgid weight (TW). Samples were dried to a constant weight at 80 °C, and the dry weight (DW) was measured. The leaf RWC was measured with FW, DW and TW, RWC (%) = [(FW − DW)/(TW − DW)] × 100^59^.

SPAD value was measured with a CCM-200 plus chlorophyll content metre (Instrumentation Consultancy Technologies). The middle of the leaves was used for the measurement to avoid the leaf vein.

### Antioxidants

SOD activity was determined according to Giannopolitis and Ries^60^. Twenty microlitres of enzyme solution was mixed with 3 ml SOD reaction solution (pH 7.8 phosphate buffer 1.5 ml, 750 mol l^−1^ NBT 0.3 ml, 130 mmol l^−1^ Met 0.3 ml, 20 mol l^−1^ FD 0.3 ml, 100 mol l^−1^ EDTA-Na_2_ 0.3 ml, distilled water 0.3 ml). The control and enzyme solution were placed for 30 min in 4000 lux light. The blank was placed in the dark and compared at 560 nm.

Peroxidase (POD) activity was determined according to Hernández *et al*.^61^. Twenty microlitres of enzyme solution was mixed with 3 ml POD reaction solution (1.4 μl guaiacol, 0.85 μl 30% H_2_O_2_ and 0.1 mol l^−1^ pH 6.0 phosphate buffer). The absorbance values were recorded once every 30 s in 470 nm.

Catalase (CAT) activity was assayed as a decrease in absorbance at 240 nm for 1 min following the decomposition of H_2_O_2_ according to Change and Maehly^62^. The reaction mixture contained 50 mM phosphate buffer (pH 7.0) and 15 mM H_2_O_2_.

Ascorbate peroxidase (APX) activity was determined according to Nakano and Asada^63^. The assay mixture consisted of 0.5 mM ASA, 0.1 mM H_2_O_2_, 0.1 mM EDTA, 50 mM sodium phosphate buffer (pH 7.0), and 0.15 ml enzyme extract.

### Chlorophyll fluorescence and photosynthesis

Chlorophyll fluorescence parameters were determined using a PAM-2500 chlorophyll fluorescence analyser (WALZ, Germany) between 9:00 and 12:00. After a 20 min dark adaptation period, the initial (F_0_), maximum fluorescence (F_m_) and ETR were determined. Maximal photochemical efficiency of PSII F_v_/F_m_ = (F_m_ − F_0_)/F_m_. The net photosynthetic rate (P_n_), stomatal conductance (G_s_), transpiration rate (Trmmol), and intercellular CO_2_ (C_i_) of the second leaf were analysed with a portable photosynthetic system (LI-6400; LI-COR, Lincoln, NE, USA). These values were measured in the middle of the leaves, which avoided the leaf vein.

### Oxidative stress

A total of 0.5 g of fresh leaves was ground in 5 ml 5% trichloroacetic acid (TCA) into a homogenate, then centrifuged at 3000 r/min for 10 min. Two millilitres of supernatant was mixed with 2 ml thiobarbituric acid (TBA), boiled in a water bath for 30 min at 100 °C, then centrifuged at 3000 r/min for 10 min after cooling. The extract absorbance was read at 450, 532 and 600 nm using a spectrophotometer^64^. A total of 0.5 g of fresh leaves was ground with 5 ml 50 mmol·l^−1^ phosphate buffer solution (pH 7.8) into a homogenate, centrifuged at 10,000 r/min for 10 min at 4 °C. The supernatant was centrifuged at 15,000 r/min for 20 min at 4 °C, and the second supernatant was used as the extract. A total of 0.5 ml extract was mixed with 0.5 ml 50 mmol · l^−1^ phosphate buffer solution and 1 ml 1 mmol·l^−1^ hydroxylamine hydrochloride. The mixture was left to stand for 1 h at 25 °C; then, 1 ml 17 mmol · l^−1^ sulfanilic acid and 1 ml 7 mmol · l^−1^ α-naphthylamine were added to the mixture and colourated for 20 min at 25 °C. The absorbance of the extract was read at 530 nm using a spectrophotometer^65^.

### Osmoprotectants

A total of 0.3 g of fresh leaves was cut into pieces and put into a graduated test tube with 5 ml distilled water. The test tube was sealed with plastic film and boiled in a water bath for 30 min (twice). The extract was filtered into 25 ml volumetric flasks; the distilled water was used to rinse the test tube, and the residues were transferred into volumetric flasks and diluted to 25 ml. A total of 0.5 ml of extract was transferred into a 20 ml graduated test tube with 1.5 ml of distilled water. The absorbance was measured at 620 nm in a UV-Vis spectrophotometer.

Proline content was determined according to Monreal *et al*.^66^ with some modifications. A total of 0.5 g of fresh leaves and 5 ml 3% sulfosalicylic acid were put into a test tube and then transferred to boiling water for 10 min. The extract was filtered into a clean test tube. Two millilitres of extract was put into a test tube with a stopper, and then 2 ml of glacial acetic acid and 2 ml of acidic ninhydrin were added. The mixture was transferred to boiling water for 30 min. Four millilitres of toluene was put into the test tube after the mixture cooling, oscillated for 30 s and held for a moment. The supernatant was transferred into a 10 ml centrifuge tube for centrifuging for 5 min at 3000 r/min. The absorbance of the supernatant was read at 520 nm using toluene as a blank.

### Determination of glutamate decarboxylase activity

Crude enzyme was extracted from cells at mid-log phase and incubated at 30 °C for 30 min in an assay reaction (total volume 200 μl) containing 50 mM potassium phosphate citrate buffer (pH 5.8), 30 mM L glutamate, 20 μM pyridoxal-5-phosphate, and 1 mM CaCl_2_. The reaction was terminated by boiling for 10 min before GABA production was determined using HPLC. GAD activity was expressed as the amount of GABA produced per minute per milligram of protein. The protein content was determined by the method of Bradford^67^ using bovine serum albumin as a standard.

### Statistical analysis

The experiment used a randomized complete block design. The data were analysed using the statistical Software Package for Social Science (SPSS) version 17.0, and all of the values were presented as the mean ± SE. Two-way analysis of variance (ANOVA) was performed on all data. Means were separated using the least significant difference (LSD) test at the 5% probability level. The use of difference between treatments implies significant difference (P = 0.05), while no difference implies no significant difference.

## Additional Information

**How to cite this article:** Wang, Y. *et al*. γ-Aminobutyric Acid Imparts Partial Protection from Salt Stress Injury to Maize Seedlings by Improving Photosynthesis and Upregulating Osmoprotectants and Antioxidants. *Sci. Rep.* **7**, 43609; doi: 10.1038/srep43609 (2017).

**Publisher's note:** Springer Nature remains neutral with regard to jurisdictional claims in published maps and institutional affiliations.

## Supplementary Material

Supplementary Data

## Figures and Tables

**Table 1:** Results of ANOVA on the effects of GABA (G) and NaCl concentration (N) on maize seedling growth, cell membrane permeability, GABA synthase activity, osmoprotectants, antioxidants and photosynthesis.

Effect	G	N	G × N	Error	Total variation
df	1	2	2	12	17
Plant height	6.26*	29.59**	0.45^ns^		
Leaf area per plant	9.16*	24.25**	0.66^ns^		
Leaf fresh weight per plant	9.69**	34.71**	0.72^ns^		
Leaf dry weight per plant	7.62*	19.78**	0.85^ns^		
Membrane damage	4.92*	8.96**	2.31^ns^		
SPAD value	4.76*	29.26**	0.53^ns^		
Relative leaf water content	9.97**	134.55**	6.72*		
Cellular reduction ability	8.82*	22.40**	0.99^ns^		
GAD	2283**	618**	170**		
SOD	65.74**	2209.79**	13.87**		
POD	20.90**	119.77**	7.32**		
CAT	13.64**	98.10**	7.22**		
APX	49.31**	258.91**	15.92**		
Proline	75.66**	169.04**	5.88*		
Soluble sugar	41.16**	247.84**	4.92*		
MDA	7.99*	134.89**	1.95^ns^		
Superoxide anion	31.70**	428.38**	10.66**		
Pn	22.93**	71.62**	0.29^ns^		
Trmmol	3.00^ns^	15.52**	0.05^ns^		
Ci	10.97**	124.09**	1.60^ns^		
Gs	21.33**	50.08**	2.53^ns^		
F_0_	27.78**	372.65**	11.94**		
F_m_	12.30**	107.27**	0.64^ns^		
F_v_/F_m_	25.20**	201.46**	4.80*		
ETR	5.59*	69.07**	0.54^ns^		

ns denotes non-significant, *P < 0.05, **P < 0.01.

**Table 2:** Plant height, leaf area, leaf fresh weight and leaf dry weight per plant for plants grown at varying salt stress for 48 h that were treated with 0.5 mM γ- aminobutyric acid (GABA) or left untreated.

Treatment	Plant height (cm)	Leaf area per plant (cm^2^)	Leaf fresh weight per plant (g)	Leaf dry weight per plant (g)
0 mM NaCl	29.3 ± 1.1ab	44.75 ± 2.41ab	0.993 ± 0.102a	0.082 ± 0.005b
150 mM NaCl	21.5 ± 1.8c	33.28 ± 1.95c	0.568 ± 0.069c	0.052 ± 0.005c
300 mM NaCl	15.7 ± 1.4d	24.16 ± 2.94d	0.375 ± 0.018d	0.039 ± 0.006c
0 mM NaCl + 0.5 mM GABA	31.6 ± 1.5a	48.32 ± 3.33a	1.124 ± 0.127a	0.097 ± 0.013a
150 mM NaCl + 0.5 mM GABA	26.9 ± 2.6b	43.14 ± 2.73b	0.857 ± 0.026b	0.079 ± 0.006b
300 mM NaCl + 0.5 mM GABA	18.6 ± 1.5 cd	30.97 ± 2.83c	0.517 ± 0.016c	0.047 ± 0.006c

Values are mean ± SE. Values with the same letters in a column are not significantly different at P = 0.05 (LSD test).

**Table 3:** Membrane damage, SPAD, relative leaf water content and cellular reduction ability for plants grown at varying salt stress for 48 h treated with 0.5 mM γ- aminobutyric acid (GABA) or left untreated.

Treatment	Membrane damage (%)	SPAD value	Relative leaf water content (%)	Cellular reduction ability (OD 485/50 mg FW)
0 mM NaCl	21.4 ± 0.76d	14.2 ± 1.17a	96.54 ± 1.05a	0.41 ± 0.04a
150 mM NaCl	27.9 ± 4.91ab	11.1 ± 0.66b	84.91 ± 4.54c	0.35 ± 0.04b
300 mM NaCl	30.3 ± 1.23a	8.5 ± 1.59c	73.03 ± 0.75d	0.24 ± 0.03c
0 mM NaCl + 0.5 mM GABA	22.4 ± 2.41 cd	14.7 ± 1.91a	94.82 ± 1.31a	0.44 ± 0.01a
150 mM NaCl + 0.5 mM GABA	23.6 ± 1.88 cd	13.1 ± 0.5a	89.02 ± 0.87b	0.41 ± 0.02a
300 mM NaCl + 0.5 mM GABA	25.7 ± 1.45bc	9.7 ± 0.75bc	75.75 ± 0.51d	0.26 ± 0.03c

Values are mean ± SE. Values with the same letters in a column are not significantly different at P = 0.05 (LSD test).

**Table 4:** Activity of enzymatic antioxidants in leaves of plants grown at varying salt stress for 48 h that were treated with 0.5 mM γ- aminobutyric acid (GABA) or left untreated.

Treatment	Superoxide dismutase (U·g^−1^)	Peroxidase (U·g^−1^·min^−1^)	Catalase (U·g^−1^·min^−1^)	Ascorbate peroxidase (U·g^−1^·min^−1^)
0 mM NaCl	52.14 ± 1.94c	13.02 ± 0.77cd	15.11 ± 0.55b	20.47 ± 1.92c
150 mM NaCl	125.25 ± 4.38b	19.79 ± 2.04b	17.36 ± 0.79b	32.14 ± 2.67b
300 mM NaCl	31.47 ± 1.75e	8.41 ± 0.45e	10.74 ± 0.67d	10.34 ± 1.85e
0 mM NaCl + 0.5 mM GABA	54.57 ± 1.49c	14.12 ± 2.55c	14.74 ± 0.84bc	21.24 ± 1.76c
150 mM NaCl + 0.5 mMGABA	144.32 ± 3.58a	27.54 ± 1.88a	21.21 ± 1.76a	45.96 ± 2.44a
300 mM NaCl + 0.5 mMGABA	41.39 ± 2.01d	10.21 ± 1.15de	12.29 ± 0.63 cd	15.88 ± 1.20d

Values are mean ± SE. Values with the same letters in a column are not significantly different at P = 0.05 (LSD test).
