# Supplementary material for: γ-Aminobutyric Acid Imparts Partial Protection from Salt Stress Injury to Maize Seedlings by Improving Photosynthesis and Upregulating Osmoprotectants and Antioxidants
Source: Sci Rep. 2017 Mar 8;7:43609. doi: 10.1038/srep43609 (PMC5341084; doi:10.1038/srep43609)
Supplement: Supplementary Data [file srep43609-s1.pdf]

γ-Aminobutyric Acid Imparts Partial Protection from Salt Stress Injury to Maize Seedlings by Improving Photosynthesis and Upregulating Osmoprotectants and Antioxidants

Yongchao Wang[1], Wanrong Gu[1, 2*], Yao Meng[1, 3], Tenglong Xie[1], Lijie Li[1], Jing Li[1, 2], Shi Wei[1, 2]

[1]College of Agriculture, Northeast Agricultural University, Harbin 150030, P.R. China. [2]The Observation Experiment Station of Ministry of Agriculture for Crop Cultivation Science in Northeast Area, Harbin 150030, P.R. China. [3]Heilongjiang Academy of Land Reclamation Sciences, Harbin 100030, P.R. China. *Correspondence and requests for materials should be addressed to W.G. (email: wanronggu@163.com)

Effect of various GABA concentration on maize seedling growth at salt stress 48 h

Treatment	Shoot fresh weight (g/plant)	Root fresh weight(g/plant)	Shoot dry weight(g/plant)	Root dry weight(g/plant)	Root length(cm/plant)	Root surface area(cm2/plant)	Root volume(cm3/plant)	The number of root tips
0 mM NaCl	1.81±0.07a	1.39±0.05a	0.427±0.004a	0.115±0.004a	310±9.53a	80.57±2.50a	2.94±0.10a	348±10.97a
150 mM NaCl	1.14±0.05d	0.94±0.07c	0.321±0.006e	0.086±0.001b	183±9.24c	48.46±3.01c	1.78±0.13c	226±9.81c
150 mM NaCl+0.25 mM GABA	1.35±0.06c	1.02±0.01bc	0.363±0.008c	0.091±0.002b	229±10.39b	60.23±2.62b	1.92±0.06c	236±10.39bc
150 mM NaCl+0.5 mM GABA	1.55±0.02b	1.29±0.02a	0.409±0.008b	0.112±0.002a	332±12.70a	75.68±4.77a	2.52±0.08b	331±15.01a
150 mM NaCl+1.0 mM GABA	1.32±0.05c	1.13±0.01b	0.351±0.005cd	0.095±0.004b	218±13.86bc	61.59±2.46b	2.38±0.17b	258±9.81b
150 mM NaCl+2.0 mM GABA	1.25±0.02cd	1.06±0.02b	0.345±0.004d	0.095±0.006b	239±16.17b	63.89±3.65a	2.56±0.09b	269±10.97b

Values are mean ±SE. Values with the same letters in a column are not significantly different at P=0.05 (LSD test).

Effect of exogenous GABA on fresh and dry weight of maize seedling root under salt stress

Treatment	Root fresh weight(g)	Root dry weight
0 mM NaCl	0.682±0.007a	0.058±0.004a
150 mM NaCl	0.549±0.016c	0.049±0.004b
300 mM NaCl	0.416±0.014d	0.036±0.002c
0 mM NaCl+0.5 mM GABA	0.721±0.012a	0.061±0.003a
150 mM NaCl+0.5 mM GABA	0.605±0.012b	0.054±0.002ab
300 mM NaCl+0.5 mM GABA	0.448±0.016d	0.039±0.002c

Values are mean ±SE. Values with the same letters in a column are not significantly different at P=0.05 (LSD test).
